# Interleukin-6 as a Prognostic Biomarker in Ruptured Intracranial Aneurysms

**DOI:** 10.1371/journal.pone.0132115

**Published:** 2015-07-15

**Authors:** Hung-Wen Kao, Kwo-Whei Lee, Chen-Ling Kuo, Ching-Shan Huang, Wan-Min Tseng, Chin-San Liu, Ching-Po Lin

**Affiliations:** 1 Department of Radiology, Tri-Service General Hospital, National Defense Medical Center, Taipei, Taiwan; 2 Department of Biomedical Imaging and Radiological Sciences, National Yang-Ming University, Taipei, Taiwan; 3 Department of Medical Imaging, Changhua Christian Hospital, Changhua, Taiwan; 4 Vascular and Genomic Center, Changhua Christian Hospital, Changhua, Taiwan; 5 Department of Neurology, Changhua Christian Hospital, Changhua, Taiwan; 6 Graduate Institute of Integrative Medicine, China Medical University, Taichung, Taiwan; 7 Institute of Neuroscience, School of Life Science, National Yang-Ming University, Taipei, Taiwan; Emory University School of Medicine, UNITED STATES

## Abstract

**Background:**

Interleukin-6 (IL-6), a proinflammatory cytokine, was found to surge in the cerebral spinal fluid after aneurysmal subarachnoid hemorrhage (SAH). We hypothesized that the plasma level of IL-6 could be an independent biomarker in predicting clinical outcome of patients with ruptured intracranial aneurysm.

**Methods:**

We prospectively included 53 consecutive patients treated with platinum coil embolization of the ruptured intracranial aneurysm. Plasma IL-6 levels were measured in the blood samples at the orifices of the aneurysms and from peripheral veins. The outcome measure was the modified Rankin Scale one month after SAH. Multiple logistic regression analyses were used to evaluate the associations between the plasma IL-6 levels and the neurological outcome.

**Results:**

Significant risk factors for the poor outcome were old age, low Glasgow Coma Scale (GCS) on day 0, high Fisher grades, and high aneurysmal and venous IL-6 levels in univariate analyses. Aneurysmal IL-6 levels showed modest to moderate correlations with GCS on day 0, vasospasm grade and Fisher grade. A strong correlation was found between the aneurysmal and the corresponding venous IL-6 levels (ρ = 0.721; *P*<0.001). In the multiple logistic regression models, the poor 30-day mRS was significantly associated with high aneurysmal IL-6 level (OR, 17.97; 95% CI, 1.51–214.33; *P* = 0.022) and marginally associated with high venous IL-6 level (OR, 12.71; 95% CI, 0.90–180.35; *P* = 0.022) after adjusting for dichotomized age, GCS on day 0, and vasospasm and Fisher grades.

**Conclusions:**

The plasma level of IL-6 is an independent prognostic biomarker that could be used to aid in the identification of patients at high-risk of poor neurological outcome after rupture of the intracranial aneurysm.

## Introduction

Intracranial aneurysms are relatively common with an overall prevalence estimated as 3.2% and an annual risk of rupture as approximately 1% [1,2]. Aneurysmal subarachnoid hemorrhage (SAH) is fatal or disabling in two-third of the cases with vasospasm being the leading culprit [3–5]. However, the CONSCIOUS trials showed equivocal effects from prevention of angiographic vasospasm [6–10]. These findings are examples of the fact that the development of the cerebral vasospasm is multifactorial and the exact pathophysiology of brain injury in SAH remains unclear. Several studies have shown that older patient age, the occurrence of vasospasm, and more SAH on admission computed tomography (CT) are commonly associated with a poor clinical outcome [11,12]. However, evaluation of vasospasm and amount of SAH requires imaging studies, such as transcranial Doppler, CT, and magnetic resonance imaging, which are sometimes challenging to be obtained particularly in complicated patients. It would be valuable to have a biochemical marker to assist in predicting clinical outcome in the early stage of SAH. In patients with SAH, the blood in the cerebrospinal fluid (CSF) space activates a series of inflammatory cascades. Several studies have shown a growing recognition that inflammation, both local and systemic, could be an important manifestation of a worsening clinical course [13,14].

Interleukin-6 (IL-6) is a pleiotropic inflammatory cytokine of low molecular weight, approximately 26 kD, with a biological half-time of less than one hour [15]. Generally, IL-6 exerts proinflammatory effects including stimulation of the growth of mature B cells and promotion of the synthesis of C-reactive protein by the liver during tissue injury of infection [15,16]. High circulating IL-6 levels are associated with various diseases, including cardiovascular disease, type 2 diabetes mellitus, cancer growth, acute cerebral ischemia, and acute brain injury [17–21]. After rupture of the intracranial aneurysm, high CSF IL-6 levels were found to associate with vasospasm [22–24]. However, the role of IL-6 in local aneurysmal microenvironment is rarely investigated. In a cohort of 13 unruptured and three ruptured intracranial aneurysm, Chalouhi et al. found no significant difference between the IL-6 levels measured within intracranial aneurysms and those measured in the femoral arteries in the unruptured subgroup [25]. To our knowledge, the clinical implication of aneurysmal IL-6 levels in patients with ruptured intracranial aneurysms remain unclear.

In this study, we aimed to measure the IL-6 levels at the orifices of intracranial aneurysms and peripheral veins and to assess their associations with clinical neurological severity and outcome. We hypothesize that high plasma IL-6 level is an independent risk factor for clinical outcome.

## Methods

### Patients

The study was reviewed and approved by the institutional ethics review board of Changhua Christian Hospital in Taiwan (ethical review code: 080106) and the written informed consent was obtained from each participant or next of kin. We prospectively studied consecutive patients with ruptured saccular intracranial aneurysms in a single medical center from 2009 to 2013. Patients were included as early as possible when endovascular embolization was initiated for treatment. Patients with a history of head trauma, brain tumors, or hematological disorders were excluded.

In admission, white blood cell count (WBC) was routinely measured for all patients. Clinical characteristics, including age, sex, pack-years of smoking, and history of hypertension and diabetes mellitus were recorded. An experienced neurologist (CSL), blinded to the IL-6 levels, evaluated the clinical severity of SAH by means of the Glasgow Coma Scale (GCS) on day 0 and by the modified Rankin Scale (mRS) on day 30. All patients suspected to have ruptured intracranial aneurysms were studied with a dual source CT system (SOMATOM Definition FLASH, Siemens Healthcare, Forchheim, Germany) before the endovascular procedure. An experienced neuroradiologist (KWL) assessed the amount of blood on the CT images of the brain according to the Fisher scale [26] and gauged the culprit aneurysms based on the distribution of the blood and findings on CT angiography.

### Endovascular procedures

As soon as the patient was stable in vital signs and transferred to our neuroangiography suite, a targeted three-dimensional rotational digital subtraction angiography of the culprit aneurysm was performed via a femoral approach using a 6-French system under general anesthesia. All angiograms were obtained on an Axiom Artis zee biplane (Siemens Medical Solution, Erlangen, Germany) neuroradiologic angiography system. The non-ionic contrast material with an average flow rate of 3 mL/sec was injected into the internal carotid artery or the vertebral artery. The same neuroradiologist evaluated the size, number, location, and morphology of the intracranial aneurysm as well as the severity of vasospasm, graded on a scale of 0 to 4 using both radiologic and clinical criteria according to the study of Yalamanchili et al. [27].

In all patients, embolization of the ruptured intracranial aneurysm was performed with platinum detachable coils immediately after the diagnostic catheter angiography. After the microcatheter was positioned at the orifice of the aneurysm under fluoroscopy and roadmapping, blood samples were drawn from the microcatheter and a peripheral vein to measure the aneurysmal and venous IL-6 levels, respectively, with commercially available enzyme-linked immunosorbent assay kits (eBioscience, San Diego, CA, USA). After the first coil or first set of coils has been deployed, systemic heparinization (intravenous bolus of 70 IU/kg of body weight, to a maximum of 5000 IU, followed by a maintenance dose of 20 IU/kg of body weight per hour, was initiated for a targeted activated coagulation time of 2 times baseline during the procedure. Coils were then sequentially delivered into the aneurysm until a solid cast was achieved. In patients with multiple aneurysms, the culprit aneurysm was decided based on the distribution of the SAH on CT and angiographic morphology of the aneurysm. The non-culprit aneurysms were treated with decisions taking into account the risk of additional endovascular procedures. For patients with wide-neck aneurysms, the endovascular procedures were assisted with stents. Adjunctive therapies were administered at the discretion of our neurosurgical team in adherence to the department guidelines, which include ventriculostomy for patients with symptomatic hydrocephalus and intravenous infusion of nimodipine in a dose of 60 mg/day during the first week and 40 mg/day in the second week, and 20 mg/day in the third week. Follow-up CT scans were performed for patients with clinical suspicion of recurrent hemorrhage or infection.

### Statistical analyses

The poor outcome was defined as an mRS score of 3 or higher on day 30. The between-group differences on variables of clinical presentation and outcome were evaluated for statistical significance with the Mann-Whitney U test for continuous variables and the Fisher’s exact test for categorical variables. Scores of clinical severity were assessed for statistically significant differences between the different locations of the aneurysm by Kruskal-Wallis tests. Receiver operating characteristic (ROC) curves were constructed to determine the optimal thresholds for the risk factors that reached *P*<0.1 in the univariate analyses to predict poor 30-day outcome. Multiple logistic regression models were analyzed to determine associations between plasma IL-6 levels and 30-day outcome after adjusting for the risk factors that reached *P*<0.1 in the univariate analyses. In the models, the odds ratios and 95% confidence intervals of the predictors were estimated.

The Spearman rank correlation coefficients between the aneurysmal IL-6 levels and other variables were computed. A 2-sided *P*<0.05 was considered significant. The statistical analyses were performed with the IBM SPSS Statistics for Mac (version 20; IBM Corp., Armonk, NY) and MedCalc for Windows (version 14.10.2; MedCalc Software, Mariakerke, Belgium).

## Results

Among 61 consecutive patients with ruptured saccular intracranial aneurysm treated in our neuroangiography suite from 2009 to 2013, 8 patients (13.1%) were excluded due to inadequate blood sampling during the emergent coil embolization (5 patients) and difficulty in determining the culprit aneurysm (3 patients). A total of 53 patients (64 aneurysms; 32 women and 21 men; mean age 54 years; range 28–82) were included for analysis. All the 53 culprit aneurysms were secured with platinum detachable coils within 24 hours of admission and within three days of symptom onset. Five patients (9.4%) died before day 30. The most common location of the ruptured aneurysm was in the anterior communicating artery (19 patients, 35.8%) followed by posterior communicating artery (17, 32.1%), middle cerebral artery (8, 15.1%), internal carotid artery (5, 9.4%), vertebral artery (3, 5.7%) and basilar artery (1, 1.9%). GCS scores on day 0 and mRS on day 30 were not significantly different between patients with different locations of the aneurysm (*P* = 0.120 and 0.122 respectively). During the follow-up period, no patient was found to have recurrent intracranial hemorrhage or infection.

The characteristics of the studied patients are detailed in Table 1. The median aneurysm size was 5 mm (range, 2–18). The 30-day outcome was significantly associated age, GCS on day 0, Fisher grade, and aneurysmal and venous IL-6 levels. The vasospasm grade was marginally higher in patients with poor 30-day outcome than those with good outcome (*P* = 0.051).

**Table 1:** Patient characteristics.

	mRS on day 30		
Characteristics	<3 (n = 27)	≥3 (n = 26)	*P*
Age, y	49.0 (44.0–56.0)	60 (44.5–70.5)	0.033
Women (%)	14 (51.9)	18 (69.2)	0.264
Diabetes (%)	2 (7.4)	1 (3.8)	1.000
Hypertension (%)	6 (22.2)	7 (26.9)	0.757
Smoking index, pack-year	0 (0–10.0)	0 (0–2.0)	0.797
GCS on day 0	15.0 (15.0–15.0)	9.0 (7.0–15.0)	<0.001
Multiple aneurysms (%)	4 (14.8)	6 (23.1)	0.501
Aneurysm size, mm	6.0 (4.0–8.0)	5.0 (4.0–7.0)	0.511
Vasospasm grade	0 (0–0)	0 (0–2.5)	0.051
Fisher grade	3.0 (1.0–3.0)	4.0 (3.0–4.0)	<0.001
WBC, 10^9^/L	10.4 (8.4–12.7)	12.5 (8.6–15.0)	0.142
Aneurysmal IL-6, pg/mL	6.8 (3.7–11.4)	19.3 (7.0–33.7)	0.001
Venous IL-6, pg/mL	13.7 (8.2–20.4)	27.2(10.0–52.7)	0.015

Data are median (interquartile range) or number (%).

Abbreviations: GCS: Glasgow Coma Scale; IL-6: interleukin-6; mRS: modified Rankin Scale; WBC: white blood cell count.

As shown in Table 2, aneurysmal IL-6 levels showed modest to moderate correlations with GCS on day 0, vasospasm grade and Fisher grade. The vasospasm and Fisher grades are modestly correlated (ρ = 0.329; *P* = 0.016). A strong correlation was found between the aneurysmal and the corresponding venous IL-6 levels (ρ = 0.721; *P*<0.001). The median aneurysmal IL-6 level was significantly lower than the corresponding venous IL-6 level (9.9 vs. 18.1 pg/mL; *P*<0.001). However, the arteriovenous gradient of the IL-6 levels was not significantly associated with GCS on day 0 or 30-day outcome (*P* = 0.481 and 0.393 respectively).

**Table 2:** Correlations between aneurysmal IL-6 level and other characteristics.

	Aneurysmal IL-6	
Characteristics	ρ	*P* *
Age	-0.079	0.574
GCS on day 0	-0.554	<0.001
Aneurysm size	-0.025	0.861
Vasospasm grade	0.286	0.038
Fisher grade	0.461	0.001
WBC	0.261	0.059
Venous IL-6	0.721	<0.001

*: Spearman's rank correlation test.

Abbreviations: GCS: Glasgow Coma Scale; IL-6: interleukin-6; WBC: white blood cell count.

Table 3 shows the ROC analyses of the risk factors for predicting 30-day neurological outcome. In the multiple logistic regression models, the poor 30-day mRS was significantly associated with high aneurysmal IL-6 level and marginally associated with high venous IL-6 level after adjusting for dichotomized age, GCS on day 0, and vasospasm and Fisher grades according to the ROC results (Table 4). Patients with high aneurysmal IL-6 levels, >14.6 pg/mL, were 17.97 times (95% CI, 1.51–214.33; *P* = 0.022) more likely to have poor neurological outcome in 30 days of aneurysmal rupture than those with lower levels.

**Table 3:** ROC analyses of the risk factors for predicting 30-day outcome.

Risk factor	Area under ROC Curve*	Sensitivity (%)^†^	Specificity (%)^†^	Cutoff Value	*P*
Age, years	0.670 (0.520–0.821)	61.5 (16/26)	77.8 (21/27)	>56	0.027
GCS on day 0	0.823 (0.716–0.930)	73.1 (19/26)	88.9 (24/27)	≤14	<0.001
Vasospasm grade	0.610 (0.502–0.719)	23.1 (6/26)	100 (27/27)	>2	0.046
Fisher grade	0.808 (0.701–0.916)	61.5 (16/26)	85.2 (23/27)	>3	<0.001
Aneurysmal IL-6, pg/mL	0.771 (0.642–0.901)	61.5 (16/26)	92.6 (25/27)	>14.6	<0.001
Venous IL-6, pg/mL	0.695 (0.549–0.841)	53.9 (14/26)	85.2 (23/27)	>27.0	0.009

*: Data in parentheses are 95% confidence intervals.

^†^: Sensitivity and specificity for predicting poor 30-day outcome. Data in parentheses are numbers used to calculate percentages.

Abbreviations: GCS: Glasgow Coma Scale; IL-6: interleukin-6.

**Table 4:** Adjusted associations between plasma IL-6 levels and poor 30-day outcome.

	mRS ≥3 on day 30	
Plasma IL-6 level	OR (95% CI)	*P* *
High aneurysmal IL-6, >14.6 pg/mL	17.97 (1.51–214.33)	0.022
High venous IL-6, >27.0 pg/mL	12.71 (0.90–180.35)	0.060

*: Adjusted for dichotomized age, Glasgow Coma Scale on day 0, and vasospasm and Fisher grades according to the receiver operating characteristic analyses.

Abbreviations: IL-6: interleukin-6; mRS: modified Rankin Scale.

## Discussion

In this study, we found that patients with high plasma IL-6 levels were associated with a significant high risk of poor outcome in 30 days after aneurysmal SAH. The aneurysmal IL-6 level modestly to moderately associated with GCS on day 0, vasospasm grade, and Fisher grade. A strong correlation existed between the aneurysmal and venous IL-6 levels with the venous IL-6 level significantly higher than the corresponding aneurysmal level.

Elevation of IL-6 level in CSF has been shown to be a reliable early biomarker for predicting vasospasm after SAH. However, its association with the outcome was found only in one study of Fassbender et al., demonstrating that increased CSF IL-6 level at day 5 was significantly associated with poor outcomes [22]. Similar to the implication of the CSF IL-6 level, our results showed that plasma levels of IL-6 could be valuable outcome predictors in patients with rupture of the intracranial aneurysm.

Patients with aneurysmal SAH generally shows increased IL-6 levels in CSF, cerebral extracellular fluid, and peripheral veins with the levels in the CSF and cerebral extracellular fluid significantly associating with ischemic deficits [28]. The finding suggested a compartmentalized inflammatory host response although the mechanism of the inflammatory reaction is unclear. In our results, a higher level of IL-6 in circulating venous blood than in local aneurysmal microenvironment may imply a role of systemic inflammatory response in addition to the localized reaction in the ruptured aneurysmal lumen.

In a study of abdominal aortic aneurysms, John et al. showed higher IL-6 in the iliac artery than in the brachial one with a gradient increasing with aneurysm diameter [29]. In contrast, the aneurysmal IL-6 level in our study was not associated with the aneurysm size. The finding may result from the relatively small size of the intracranial aneurysm compared to the size of the aortic aneurysm. Therefore, in our study, the correlation between plasma IL-6 levels and the size of the aneurysm is less likely to be significant and the plasma IL-6 levels more likely reflected the degree of inflammation than the aneurysm size.

The strength of our study was the direct measurement of IL-6 at the orifice of the intracranial aneurysm with simultaneous blood sample drawn from the peripheral vein to elucidate the association between circulating IL-6 levels and clinical outcome. The limitation of our study was basically due to the fact that the included subjects were from only one local medical center. Patients undergoing surgical clipping of the aneurysm were not included in our study to limit the confounding influence of the surgery. We did not longitudinally measure the plasma IL-6 during follow-up and aimed to find early parameters in three days to predict patient outcomes. Another potential limitation was that we did not routinely perform follow-up CT to fully assess potential confounding factors, such as recurrent hemorrhage, hydrocephalus, and delayed cerebral ischemia. However, in a study of McMahon et al., initial venous IL-6 levels after aneurysmal SAH is not associated with delayed cerebral ischemia [30]. Furthermore, the occurrence of delayed cerebral ischemia might not be the cause of poor outcomes [31].

## Conclusions

Plasma IL-6 levels could be an early independent outcome predictor for patients with ruptured intracranial aneurysm. Patients with high plasma IL-6 levels in the first three days of aneurysm rupture are more likely to have a poor outcome in 30 days.
